# Decrystallization of Crystals Using Gold “Nano-Bullets” and the Metal-Assisted and Microwave-Accelerated Decrystallization Technique

**DOI:** 10.3390/molecules21101388

**Published:** 2016-10-18

**Authors:** Nishone Thompson, Zainab Boone-Kukoyi, Raquel Shortt, Carisse Lansiquot, Bridgit Kioko, Enock Bonyi, Salih Toker, Birol Ozturk, Kadir Aslan

**Affiliations:** 1 Department of Chemistry, Morgan State University, 1700 East Cold Spring Lane, Baltimore, MD 21251, USA; nitho9@morgan.edu (N.T.); zaboo2@morgan.edu (Z.B.-K.); rasho4@morgan.edu (R.S.); calan1@morgan.edu (C.L.); brkio1@morgan.edu (B.K.); enbon1@morgan.edu (E.B.); salih.toker@morgan.edu (S.T.); 2 Department of Physics and Engineering Physics, Morgan State University, 1700 East Cold Spring Lane, Baltimore, MD 21251, USA; birol.ozturk@morgan.edu

**Keywords:** metal-assisted and microwave-accelerated evaporative de-crystallization, emblation microwave, gout, uric acid, l-alanine, gold nanoparticles

## Abstract

Gout is caused by the overproduction of uric acid and the inefficient metabolism of dietary purines in humans. Current treatments of gout, which include anti-inflammatory drugs, cyclooxygenase-2 inhibitors, and systemic glucocorticoids, have harmful side-effects. Our research laboratory has recently introduced an innovative approach for the decrystallization of biological and chemical crystals using the Metal-Assisted and Microwave-Accelerated Evaporative Decrystallization (MAMAD) technique. In the MAMAD technique, microwave energy is used to heat and activate gold nanoparticles that behave as “nano-bullets” to rapidly disrupt the crystal structure of biological crystals placed on planar surfaces. In this study, crystals of various sizes and compositions were studied as models for tophaceous gout at different stages (i.e., uric acid as small crystals (~10–100 μm) and l-alanine as medium (~300 μm) and large crystals (~4400 μm). Our results showed that the use of the MAMAD technique resulted in the reduction of the size and number of uric acid and l-alanine crystals up to >40% when exposed to intermittent microwave heating (up to 20 W power at 8 GHz) in the presence of 20 nm gold nanoparticles up to 120 s. This study demonstrates that the MAMAD technique can be potentially used as an alternative therapeutic method for the treatment of gout by effective decrystallization of large crystals, similar in size to those that often occur in gout.

## 1. Introduction

Gout is a painful crystal deposition disease with increasing prevalence throughout numerous regions of the world. Gout affects approximately 8.3 million individuals in the United States of America, and it is caused primarily by elevated serum uric acid levels, also known as hyperuricemia [1]. A diagnosis of acute gout is made when serum uric acid levels are above the range of 5.7 mg/dL to 7.0 mg/dL. Chronic gout is diagnosed when acute gout is left untreated, and uric acid crystals accumulate in the form of monosodium urate monohydrate in the joints and soft tissue forming tophi [1]. Uric acid, the by-product of purine metabolism, accumulates when excess purine-rich foods are catabolized during digestion [2,3]. Uric acid is released into the blood serum by cells that undergo apoptosis soon after the release of the acid [4]. During normal metabolism, released uric acid is processed by the kidneys and excreted in urine. Gout forms when uric acid levels exceed the excretory capacity of the kidneys or if the kidney is functionally impaired. Uric acid that remains in the blood precipitates and nucleates to form monosodium urate crystals and the aggressive deposition in the synovial fluid of the joints [4,5]. The progression of the disease leads to accumulation of crystals in joints and subsequent formation of hard masses called tophi, which is indicative of chronic gout [6].

The treatment for gout and its symptoms include a change in dietary habits, which can be considered a long-term preventative treatment of hyperuricemia. Additionally, nonsteroidal anti-inflammatory drugs (NSAIDs), cyclooxygenase-2 (COX-2) inhibitors, colchicine, or systemic glucocorticoids are administered for short-term treatment of chronic gout [7,8]. Studies have shown that drug therapies for gout cause adverse side-effects. NSAIDs used for acute gout have been connected to side-effects, such as gastrointestinal toxicity, renal toxicity or gastrointestinal bleeding [7]. COX-2 inhibitors, once thought to present fewer side effects in comparison to NSAIDs, are linked to an increased risk of cardiovascular disease development [8,9]. Colchicine, a known toxin, leads to multiple organ failures, convulsions, coma, and death [10]; glucocorticoid treatments are linked to osteoporosis, myopathy, avascular necrosis and cardiovascular diseases [11]. When gout progresses to chronic stages, invasive surgery becomes necessary. Due to the current limitations of therapeutic treatments available for gout, an alternative and noninvasive method of treatment is necessary. Thus, the aim of an innovative treatment will focus on the depletion of accumulated crystal in the joints and the reduction of systemic uric acid.

The use of microwaves has been explored for medical care purposes from the diagnosis of the various type of cancers to the ablation of affected tissues [12,13]. During tumor ablation, a pen tipped or needle shaped applicator is used to deliver medical microwaves to the tissues. Since the dielectric properties of water are different than the tissues and water cannot adjust to the speed of the microwaves, the rotation of water molecules results in friction and heat and induces necrosis in tumor cells [12].

The Aslan Research Group has recently introduced a novel technique that focused on the decrystallization of crystals, called the Metal-Assisted and Microwave-Accelerated Decrystallization (MAMAD) technique with the combined use of metal colloids (silver and gold) and microwave heating as a potential treatment for gout [14,15]. In our preliminary studies, uric acid crystals were decrystallized on an unmodified glass surface in the presence of gold colloids and with microwave heating using a conventional microwave oven. There was a reported 60% decrease in the number of the uric acid crystals in the sample after 30 min [14]. Further studies were conducted to examine the effectiveness of the MAMAD technique on a glass platform modified with collagen and in the presence of synovial fluid to mimic biological mediums found in an actual bone joint. This study evaluated the effect of silver colloids, physical stability of gold and silver colloids in solution, and the temperature changes associated with the presence of gold or silver colloids in the solution [15]. Our results indicated that gold colloids were more suitable than silver colloids due to their superior physical stability during intermittent microwave heating. Therefore, they were more effective in the reduction of the size and number of uric acid crystals in solution with the use of the MAMAD technique. We hypothesize that the MAMAD technique works by increasing the kinetic energy of the gold nanoparticles in solution and slightly increasing the temperature of the solution due to microwave heating [15]. The increase in kinetic energy increases the number of collisions of gold nanoparticles with crystals and results in the breakdown of the uric acid crystals. An increase in solution temperature causes an increase in the solubility of crystals in solution.

In this paper, our objective is to demonstrate that the MAMAD technique can be used for the decrystallization of crystals of various sizes with a medical microwave to evaluate its potential as a noninvasive method for the treatment of crystal deposition diseases in the future. Decrystallization of crystals of varying sizes and composition was carried out with the use of a low power medical microwave (variable power up to 20 W, 8 GHz, solid state microwave source). The model crystals that we used are small uric acid crystals (size ~ 10–100 µm), medium l-alanine (size ~ 300 µm) and large l-alanine (size 4300 µm) crystals. Medium and large l-alanine crystals are employed as representative of the formation of tophi by either the accumulation of uric acid or calcium products in the body. Moreover, this study differs from our previous efforts where we have now used a medically approved solid-state microwave source and an applicator tip, rather than an impractical conventional microwave oven. The medical microwave is designed for the thermal ablation of tissue tumors formed in cancer with the use of heat supplied by the microwave energy, which functions by supplying localized dielectric heating to cause controlled destruction of tissue. We observed a decrease in the size and number of uric acid and the size of the l-alanine crystals by >40% when heated for intermittent periods between 60 s and 120 s with the MAMAD technique. Evaporation time, size of crystals, and concentration of crystals were found to be important factors that determine the efficiency of the MAMAD technique.

## 2. Results

### 2.1. Decrystallization of Uric Acid Crystals

**Figure 1:**
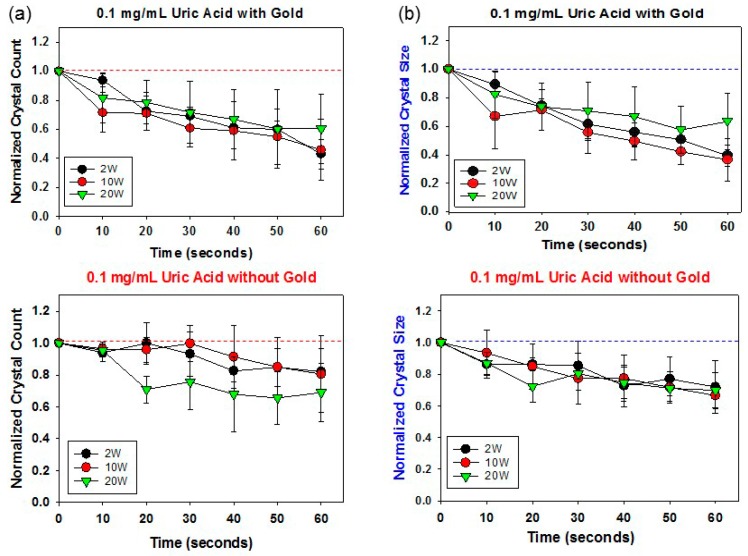
Normalized crystal (**a**) count and (**b**) size retention rate of uric acid crystals in the presence of gold nanoparticles during 60 s of microwave heating. The initial concentration of uric acid crystals was set to 0.1 mg/mL.

Figure 1 shows the normalized crystal count and size retention rates of small uric acid crystals on iCrystal plates heated at various microwave power levels (2 W, 10 W, and 20 W) with gold nanoparticles and without gold nanoparticles (a control experiment). The normalized retention rates are reflective of the efficacy of the MAMAD technique, and are calculated by dividing the total number of crystals or total area of crystals at a given time by the initial number or entire area of crystals. An increase in values beyond 1.0 is indicative of the uric acid crystals precipitating from solution due to re-crystallization. The overall decrease in normalized retention indicates that the uric acid crystals were decrystallized and demonstrates the extent of the effectiveness of the MAMAD technique.

Figure 1a shows that, for the small uric acid crystals at an initial concentration of 0.1 mg/mL, all three microwave power levels are effective in the reduction of the uric acid crystal count in comparison to their relative controls (labeled as uric acid without gold). In the presence of gold nanoparticles, after 60 s of exposure to microwaves, an average 57% and 54% decrease in the number of uric acid crystals was observed when exposed to 2 W and 10 W power levels, respectively. In the control experiment carried out at 2 W, the number of uric acid crystals was reduced by 18%, and, in the 10 W control experiment, there was a 19% reduction in the crystal count.

In the presence of gold nanoparticles and microwave heating at 20 W, the average count for uric acid crystals showed a decrease of ~40% after 60 s. Under identical experimental conditions, control samples without gold nanoparticles exposed to 20 W microwave power showed a decrease of 31% in the number of uric acid crystals, which implies that there is a 9% difference between the control and the MAMAD technique.

According to Figure 1b, normalized crystal size is decreased effectively when exposed to 2 W and 10 W microwave power levels. In the presence of gold nanoparticles and microwave heating at 10 W, there was an average decrease of 63% in the uric acid crystal size, which is significantly higher than only a 33% reduction in uric acid crystal size in the control samples after 60 s. Uric acid crystals in the presence of gold nanoparticles and microwave heated at 2 W showed an average decrease of 61% in the total surface area. In control samples exposed to microwave heating at 2 W, there was only a 28% decrease in crystal size after 60 s. Uric acid crystal samples in the presence of gold nanoparticles exposed to 20 W microwave power showed a 37% decrease in crystal size after 60 s. The control samples indicated a 31% average crystal size reduction. Detailed observation of data presented in Figure 1 revealed that after 20 s of microwave exposure, the uric acid crystals in the presence of gold nanoparticles had similar efficiency at all power levels. However, at microwave heating times exceeding 20 s, the efficiency of the MAMAD technique varied when exposed to different power levels. Similarly, after 10 s of exposure to microwave heating, the control experiments indicated similar efficacy at all energy levels. In the control experiments without gold nanoparticles, change in normalized crystal count and size leveled off after 10 s of microwave heating, which can be attributed to the absence of gold nanoparticles. Based on these observations, a 20 W power level was considered the least efficient for the decrystallization of the uric acid crystals at a concentration of 0.1 mg/mL. Therefore, the microwave power levels of 2 W and 10 W were deemed to be most efficient in the reduction of crystal size and number of uric acid crystals at a concentration of 0.1 mg/mL.

Additional experiments were carried out under identical conditions for uric acid crystals with an initial concentration of 0.2 mg/mL (Figure S1, Supplementary Materials), where the use of microwave heating at 10 W and 20 W yielded up to a 60% decrease in the crystal count and size. However, Figure S1b reveals that all power levels are equally effective in the reduction in crystal size as compared to their relative control experiments. These observations imply that the initial of the concentration of the uric acid crystals dictates the power level that is effective for the application of the MAMAD technique. Microwave heating at 10 W was found to be the most consistent effective power level for the decrystallization of 0.1 mg/mL and 0.2 mg/mL initial uric acid concentrations. Additionally, the presence of gold nanoparticles becomes more significant in the decrystallization of crystals, as the concentration of uric acid crystals in solution increases. The control experiments (Figure S1) show a less significant reduction in both crystal size and count for the uric acid crystals at the concentration of 0.2 mg/mL in comparison to those at 0.1 mg/mL. Moreover, microwave heating is essential for decreased crystallinity of uric acid crystals. Figure S2 reveals that in the absence of microwave heating and gold nanoparticles, there is insignificant change in the size and count (<10%) after 120 s, which provides additional evidence for the need for a combined use of gold nanoparticles and microwave heating in the decrystallization of uric acid crystals using the MAMAD technique.

#### Image Analysis of Small Uric Acid Crystals

**Figure 2:**
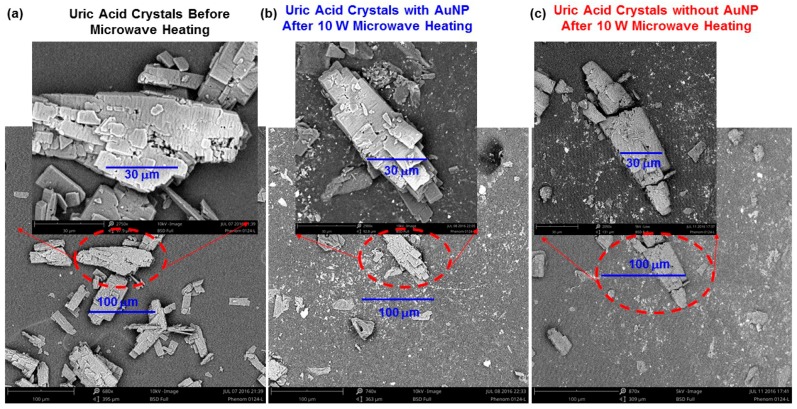
Scanning electron microscope (SEM) images of uric acid crystals (0.2 mg/mL) (**a**) before and (**b**) after microwave heating for 60 s in the presence of gold nanoparticles (i.e., the Metal-Assisted and Microwave-Accelerated Evaporative Decrystallization (MAMAD) technique) and (**c**) after microwave heating in the absence of gold nanoparticles (a control experiment) at 10 W. Scale bars are 100 μm (large image) and 30 μm (small image).

Scanning electron microscope (SEM) and optical images of the uric acid crystals on iCrystal plates after exposure to 2–20 W of microwave heating in the presence (labeled as MAMAD) and absence of gold nanoparticles (labeled as control) are given in Figure 2 and Figures S3–S6, respectively. Optical images of small uric acid crystals showed that when uric acid crystals are treated with gold nanoparticles and exposed to microwave heating, the size and number of crystals decrease significantly. For example, the use of the MAMAD technique resulted in a reduction in the number of uric acid crystals from 52 crystals at *t* = 0 s to 24 crystals at *t* = 60 s (for 10 W, see Figure S3). Similar observations in crystal count were made for uric acid crystals exposed to 2 W and 20 W (Figures S4–S6 in Supplementary Materials).

In the control experiments, microwave heating of uric acid crystals in the absence of gold nanoparticles did not result in a significant decrease in the number of uric acid crystals (Figures S3–S6, Supplementary Materials). To further investigate the effect of the combined use of gold nanoparticles and microwave heating on the decrystallization of uric acid crystals, SEM images of the uric acid crystals before microwave heating and after microwave heating with and without gold nanoparticles were obtained and shown in Figure 2. Uric acid crystals before microwave heating appear to have well-defined crystal shapes (~10–100 µm in size), and larger uric acid crystals appear with fractures on their surface as large as 10 µm (Figure 2a). After the exposure of uric acid crystals to microwave heating in the presence of gold nanoparticles for 60 s (Figure 2b), numerous crystals smaller than ~1 µm appear on and around the larger uric acid crystals, which can be attributed to the use of the MAMAD technique. On the other hand, microwave heating of uric acid crystals in the absence of gold nanoparticles (Figure 2c) resulted in the formation of numerous crystals smaller than 1 µm appearing on and around the larger uric acid crystals, similar but at a lesser extent than those observed in Figure 2b. These observations demonstrate that the MAMAD technique can be used to effectively decrystallize uric acid crystals with a medical microwave and gold nanoparticles. 

### 2.2. Decrystallization of Medium l-Alanine Crystals (~300 µm)

**Figure 3:**
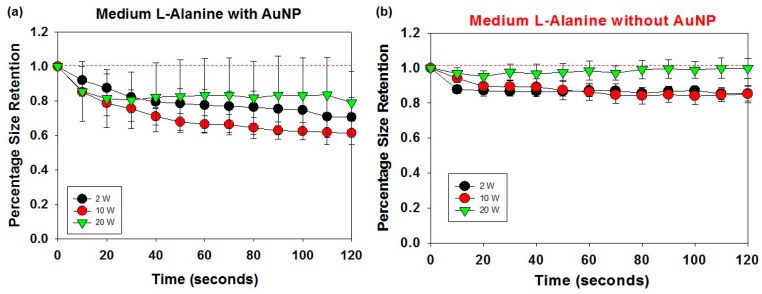
Normalized size retention rate of medium-sized l-alanine crystals (initial size = 300 μm) after 120 s of microwave heating (**a**) in the presence of gold nanoparticles and (**b**) in the absence of gold nanoparticles (control).

Figure 3 shows the normalized crystal size retention rates for medium l-alanine crystals (~300 µm) in the presence and absence of gold nanoparticles (control) exposed to 2–20 W of microwave heating for 120 s. The normalized size retention rates were calculated using the same method employed for calculation of the normalized retention rates of uric acid crystals. Microwave heating time for the decrystallization of l-alanine crystals was twice the length of time as that employed for the uric acid crystals due to the difference in sizes of the l-alanine and uric acid crystals. Figure 3a reveals that 10 W was the most effective microwave power level for the decrystallization of the medium l-alanine crystals in the presence of gold nanoparticles: after 120 s of microwave heating at 10 W, there was about a 40% decrease in the size of l-alanine crystals. When microwave power levels of 2 W and 20 W were used in the presence of gold nanoparticles, the size of the l-alanine crystals were reduced by an average of 29% and 21%, respectively. Figure 3b shows the summary of the results for control experiments, where l-alanine crystals were exposed to microwave heating in the absence of gold nanoparticles: the overall decrease in the size of l-alanine crystals exposed to 2 W and 10 W microwave power levels was approximately 15%, while a 1% decrease was observed for l-alanine crystals heated at 20 W. The reduction in size observed for the l-alanine in control experiments can be attributed to the partial dissolution of the crystal due to increase in the temperature of the solvent. It is important to note the possibility of re-crystallization of l-alanine during microwave heating: as l-alanine molecules are cleaved from the crystal structure and dissolved in solution due to increase temperature of the solvent, intermittent microwave heating can result in a heating/cooling cycle, which, in turn, can result in re-crystallization of l-alanine on the existing crystal surface. In another control experiment, where both gold nanoparticles and microwave heating are omitted from the experiment, the size of medium l-alanine crystals in deionized water was reduced by 10%, which can be attributed to dissolution of the crystals at room temperature (Figure S7 in Supplementary Materials).

#### Image Analysis of Medium l-Alanine Crystals (~300 µm)

**Figure 4:**
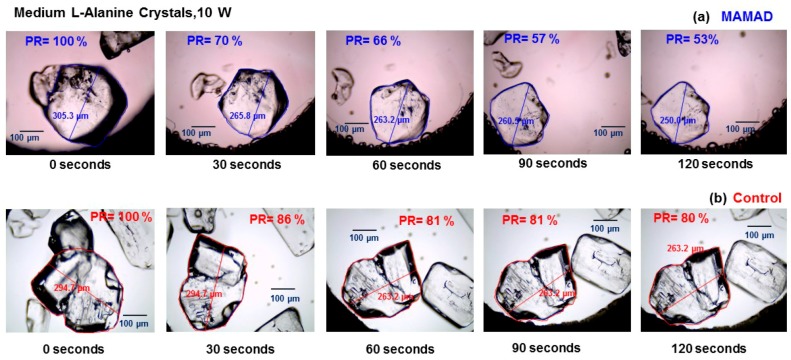
Optical images of medium-sized l-alanine crystals (initial size = 300 μm) during 10 W microwave heating for 120 s (**a**) in the presence of gold nanoparticles (i.e., the MAMAD technique); and (**b**) in the absence of gold nanoparticles (control). PR = Percent Retention and refers to the ratio of the area of the crystal to the initial area of the crystal as outlined.

**Figure 5:**
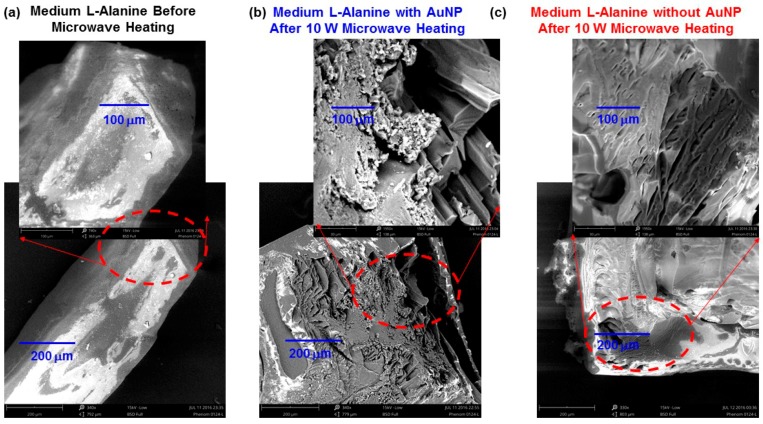
SEM images of medium l-alanine crystals (**a**) before and (**b**) after microwave heating for 120 s in the presence of gold nanoparticles (i.e., the MAMAD technique) and (**c**) after microwave heating in the absence of gold nanoparticles (control) at 10 W. Scale bars are200 μm (large image) and 100 μm (small image).

Figure 4 shows optical images of the medium (~300 µm) l-alanine crystals in the presence (i.e., the MAMAD technique) and absence of gold nanoparticles (i.e., control) during intermittent microwave heating (10 W) for 120 s. Figure 4a shows that the size (assessed by the surface area) and the length of the medium l-alanine crystals was reduced by 47% and from 305 µm to 250 µm with the application of the MAMAD technique, respectively. In a control experiment, microwave heating of medium l-alanine crystals in the absence of gold nanoparticles for 120 s resulted in 20% reduction in crystal size, and the length of medium l-alanine crystal was decreased from 295 µm to 263 µm (Figure 4b). It is important to note that all experiments were repeated at least three times to demonstrate the repeatability of the MAMAD technique and, as an example, the results for two other experiments for the decrystallization of l-alanine crystals are provided in the Supplementary Materials (Figures S8 and S9), all yielding similar results. To further investigate the effect of the combined use of gold nanoparticles and microwave heating on the decrystallization of l-alanine crystals, SEM images of the medium l-alanine crystals before microwave heating and after microwave heating with and without gold nanoparticles were obtained and shown in Figure 5. As expected, l-alanine crystals appear to have well-defined surfaces before their exposure to microwave heating (Figure 5a). Microwave heating of medium l-alanine crystals in the presence of gold nanoparticles (i.e., the MAMAD technique) results in significant damage to the crystal structure, where large and irregular-shaped cracks appear on the crystal surface. As compared to the use of the MAMAD technique, microwave heating of medium l-alanine crystals in the absence of gold nanoparticles results in a lesser extent of damage to the crystal structure, where the narrow cracks and minor surface ablation are observed. These observations provide direct evidence that the MAMAD technique can be used to decrystallize l-alanine crystals at the size of several hundred micrometers in 120 s.

### 2.3. Decrystallization of Large l-Alanine Crystals (~4300 µm)

**Figure 6:**
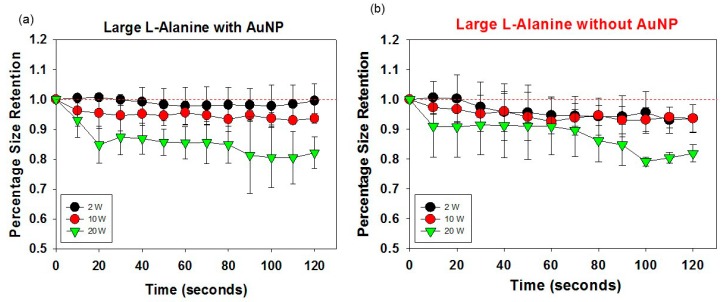
Normalized size retention rate of large l-alanine crystals (initial size = 4300 μm) after 120 s of microwave heating (**a**) in the presence of gold nanoparticles and (**b**) in the absence of gold nanoparticles (control).

**Figure 7:**
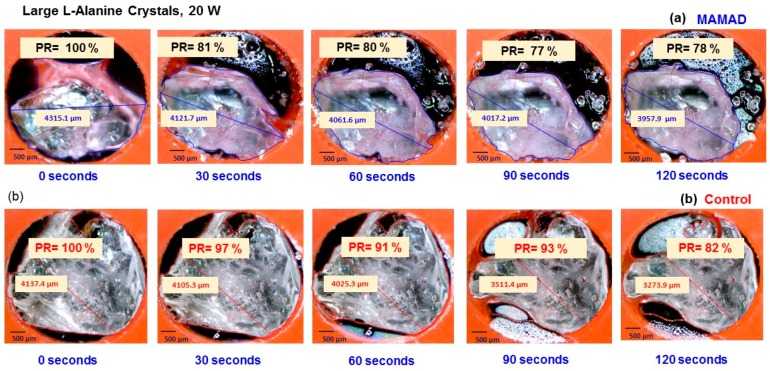
Optical images of Large l-alanine crystals on 2D poly(methyl)methacrylate PMMA platforms (**a**) with gold nanoparticles (**b**) and without gold nanoparticles at 20 W. PR = Percentage Retention.

After the successful demonstration of the use of the MAMAD technique for the decrystallization of l-alanine crystals ~300 μm in size, we investigated whether even larger l-alanine crystals (~4300 µm) can be decrystallized with the MAMAD technique. These results are given in Figure 6 and Figure 7, Figures S10 and S11 (Supplementary Materials). Figure 6 shows the normalized size retention of large l-alanine crystals (~4300 µm) in the presence and absence of gold nanoparticles (control) exposed to 2–20 W of microwave heating for 120 s.

Figure 6a shows that the size of large l-alanine crystals exposed to microwave heating in the presence of gold nanoparticles was reduced by 18%, 7% and 1% for power levels of 20 W, 10 W, and 2 W, respectively. Figure 6b reveals that the size of large l-alanine crystals exposed to microwave heating without gold nanoparticles was reduced by 18%, 6% and 7% for power levels of 20 W, 10 W, and 2 W, respectively. These results indicate that microwave heating of large l-alanine crystals in the presence and absence of gold nanoparticles yield comparable results. The reduced effectiveness of the MAMAD technique for the decrystallization of large l-alanine crystals was likely due to several factors: size of the crystal, size of the gold nanoparticles, amount of gold nanoparticle solution added (10 μL) and evaporation of the solvent during microwave heating. It is important to note that the size of the gold nanoparticles used in this study (20 nm) is significantly smaller than the size of the l-alanine crystals (~4300 µm). In addition, 10 μL of gold nanoparticles cover only the top region of the l-alanine crystals. Therefore, the collisions of the gold nanoparticles are expected to only occur when the solvent is present. After 30 s of microwave heating at 20 W, the solvent quickly evaporates and the reduction in solution volume limits the movement of gold nanoparticles in solution. Subsequently, the collisions of gold nanoparticles with l-alanine crystals are significantly reduced. The dissolution of large l-alanine crystals in deionized water in the absence of gold nanoparticles at room temperature was negligible (Figure S7 in Supplementary Materials).

#### Optical Image Analysis of Large l-Alanine Crystals (~4300 µm)

Figure 7 shows optical images of the large (~4300 µm) l-alanine crystals in the presence (i.e., MAMAD) and absence of gold nanoparticles (i.e., control) during intermittent microwave heating (20 W) for 120 s. Figure 7a shows that about a 19% reduction in the size of large l-alanine crystals was observed in 30 s of microwave heating in the presence of gold nanoparticles. Figure 7a also shows a decrease in crystal length from ~4315 µm to ~3956 µm with a combined reduction in the size of ~22% in 120 s. When large l-alanine crystals are exposed to microwave heating in the absence of gold nanoparticles (control, Figure 7b), a decrease in crystal length from ~4137 µm to ~3273 µm and a total size decrease of ~18% were observed. Figures S10 and S11 demonstrated that microwave heating of large l-alanine at microwave power levels 2 W and 10 W resulted in <5% decrease in size and length of l-alanine crystals, and are subsequently, deemed ineffective in decrystallization of large l-alanine crystals.

**Figure 8:**
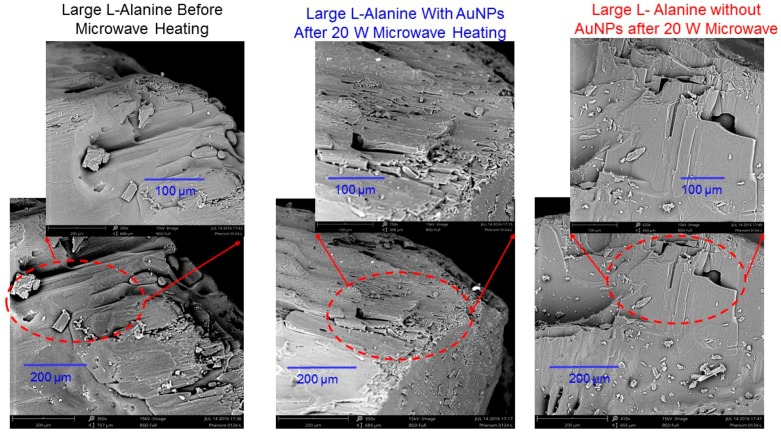
SEM images of large l-alanine crystals (4300 μm) (**a**) before and (**b**) after microwave heating for 120 s in the presence of gold nanoparticles (i.e., the MAMAD technique) and (**c**) after microwave heating in the absence of gold nanoparticles (control) at 20 W. Scale bars are 200 μm (large image) and 100 μm (small image).

To further investigate the effect of the combined use of gold nanoparticles and microwave heating on the decrystallization of l-alanine crystals, SEM images of the large l-alanine crystals before microwave heating and after microwave heating with and without gold nanoparticles were obtained and shown in Figure 8. Large l-alanine crystals appear to have well-defined surfaces before their exposure to microwave heating (Figure 8a). Microwave heating of large l-alanine crystals in the presence of gold nanoparticles results in damage to the crystal structure, where irregular-shaped cracks and smaller crystals appear on the crystal surface. As compared to the use of the MAMAD technique, microwave heating of large l-alanine crystals in the absence of gold nanoparticles results in a lesser extent of damage to the crystal structure, where the narrow cracks and minor surface ablation are observed. These observations provide direct evidence that the MAMAD technique can be used to decrystallize large l-alanine crystals at the size of several millimeters in 120 s.

**Figure 9:**
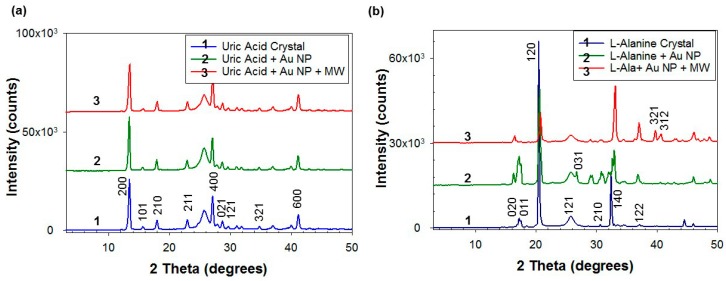
Powder X-ray diffraction (XRD) patterns of (**a**) uric acid before the MAMAD technique is applied (labeled as uric acid crystal), after incubation with gold nanoparticles (uric acid + Au NP) and after the MAMAD technique is applied (uric acid + Au NP + MW) and (**b**) medium l-alanine crystals before the MAMAD technique is applied (labeled as l-alanine crystal), after incubation with gold nanoparticles (Alanine + Au NP) and after the MAMAD technique is applied (Ala. + Au NP + MW).

It is also important to characterize the uric acid crystals and l-alanine crystals powder X-ray diffraction (XRD) analysis to determine the effect of microwave heating on the polymorphism of these crystals and/or potential conversion of the crystalline structure into an amorphous material. Figure 9a shows that the crystallinity of uric acid remained unchanged in the presence of gold nanoparticles at room temperature and after exposure to microwave heating (i.e., the MAMAD technique), which was assessed by the presence of identical diffraction patterns (i.e., no broadening of peaks and appearance/disappearance of additional peaks). Figure 9b shows that the diffraction patterns for l-alanine were maintained after the addition of gold nanoparticles (l-alanine + Au NP). These observations imply that both uric acid crystals and l-alanine crystals can be broken down into smaller crystals and decrystallized by the use of MAMAD technique.

## 3. Discussion

### The Use of the MAMAD Technique for Decrystallization of Crystals

It is important to note that medical microwaves are used in hyperthermia therapy, inflammation therapy, and in the diagnosis and treatment of cancer [16,17,18]. The choice of microwave source for the heating of tissue samples depends on the frequency of the microwaves and the penetration depth of the waves through dielectric materials within the skin: microwaves with shorter wavelength can penetrate deeper into the skin than the microwaves with longer wavelengths [19,20]. For example, microwaves at 915 MHz and 2.45 GHz are suited for large volume ablation [16], while the use of higher frequencies is suitable for treatments such as skin cancer, ablation of heart tissue to treat arrhythmia, corneal ablation for vision correction, spinal nerve ablation to treat back pain, varicose vein treatment and verrucae treatment, etc. Higher frequency microwave treatments in the range 5.8–10 GHz can create shallow penetration of energy and are therefore ideal for near surface based treatments, such as the MAMAD technique.

The combined use of microwave and metal nanoparticles in solution (i.e., the MAMAD technique) was proven and tested to be an effective method for the reduction of the crystallinity of uric acid and l-alanine crystals. In the MAMAD technique, microwave heating of the crystals in the presence of solution with gold nanoparticles results in the increase in the kinetic energy of the gold nanoparticles [21]. The increase in kinetic energy of the gold nanoparticles causes an increase in the collisions between the gold nanoparticles and l-alanine or uric acid crystals. In addition, a slight increase in the temperature of the solvent and crystal can be observed. Subsequently, increased collisions between the gold nanoparticles and increased solvent temperature results in the rapid decrystallization of the crystals, when the MAMAD technique is employed. Several factors, which include solvent evaporation, microwave heating power and time, and the size and amount of both gold and organic crystals, were found to influence the rate of decrystallization using the MAMAD technique.

Microwave heating at 10 W in the presence of gold nanoparticles was found to be the most efficient power level for the decrystallization of uric acid crystals and medium l-alanine crystals. In the MAMAD technique, evaporation of the solvent plays an important role in the decrystallization process. After the commencement of microwave heating, the volume of solvent starts to decrease, and the movement of gold nanoparticles in solution is hindered. The largest extent of evaporation was observed for microwave heating at 20 W. The use of the MAMAD technique resulted in the decrease in the size and number of the uric acid and l-alanine crystals and the evaporation of the solvent promoted the precipitation of crystals from solution, which account for the appearance of smaller uric acid crystals on the iCrystal plates. 

The amount of time necessary for effective decrystallization of crystals was found to be dependent on the initial size of the crystals. Longer microwave heating periods were needed for the medium and large l-alanine crystals as compared to smaller uric acid crystals. The MAMAD technique proved more efficient for the decrystallization of uric acid crystals and the medium sized l-alanine crystals, though it was less efficient for the large l-alanine crystals under identical experimental conditions. At a particular crystal size (i.e., large l-alanine crystals, 4300 μm), the use of 20 nm gold nanoparticles is no longer effective in the decrystallization of large l-alanine crystals due to the small size of gold nanoparticles and the evaporation of the solvent. The limitation of the use of the MAMAD technique for the large crystals may be alleviated by increasing the size of gold nanoparticles and increasing the amount of solvent available for the gold nanoparticles to move around in solution. These studies are underway and will be reported in due course.

Furthermore, the initial number of uric acid crystals was found to affect the optimal power level and the efficiency of the MAMAD technique. When the number of uric acid crystals were increased from within the range of 52–76 to 150–180 crystals, effective microwave power levels also varied: the use of microwave heating at 2 W and 10 W were most effective for the reduction of uric acid crystal count and size at a concentration of 0.1 mg/mL. The use of microwave heating at 10 W and 20 W were efficient for uric acid crystals at concentrations of 0.2 mg/mL. Our observations suggested that as the number of crystals in solution increases, the 2 W power level becomes less efficient in the decrystallization of uric acid crystals, which may be attributed to the inability of microwave heating at 2 W to provide adequate kinetic energy to the gold nanoparticles to disrupt the structure of the uric acid crystals. It is interesting to note the observation shown in Figure S1b, where microwave heating at 2 W was of similar efficacy in the reduction of crystal sizes as compared to microwave heating at 10 W and 20 W; however, the use of 2 W yielded less reduction in the number of crystals, and, subsequently, microwave heating of uric acid crystals at 2 W was deemed inefficient for decrystallization purposes. On the other hand, microwave heating of uric acid crystals at 10–20 W in the presence of gold nanoparticles can be considered efficient for decrystallization of uric acid crystals. 

It is also necessary to comment on the effect of temperature on decrystallization. Real-time temperature measurements of the solvent during microwave heating reveal a slight increase (up to 2 °C) at the end of microwave heating 60 s for uric acid crystals and 120 s for l-alanine crystals. Although an increase in the temperature in the solvent contributes to the dissolution of crystals, increased collisions between the gold “nano-bullets” and “target” crystals due to focused microwave heating is found to be the major factor in decrystallization of uric acid and l-alanine crystals. Further studies will cover the use of larger gold nanoparticles and larger uric acid crystal masses to investigate the efficiency of the use of the MAMAD technique for the decrystallization of crystals in similar size and number observed in chronic gout patients. These studies are also underway and will be reported in due course.

## 4. Materials and Methods 

### 4.1. Materials

l-Alanine, uric acid, gold nanoparticle solution (diameter = 20 nm, optical density = 1), glass coverslips, 20 mL glass vials, stir bars and thermometers were purchased from Sigma-Aldrich (St. Louis, MO, USA). Deionized water was obtained from Millipore Direct Q3 UV apparatus (Billerica, MA, USA). Adhesive silicone isolators with 21-well capacity (depth = 2.0 mm, diameter = 4.5 mm with a capacity of 30 µL, and diameter = 3.5 mm with a capacity of 10 µL) were designed by the Aslan Research Group and manufactured by Grace BioLabs (Bend, OR, USA). Poly(methyl)methacrylate (PMMA) discs (5 cm diameter) were purchased from McMaster-Carr (Chicago, IL, USA). The iCrystal plates were created by combining the PMMA discs and the adhesive silicone isolators. Adhesive silicon isolator was carefully attached to the PMMA platform and gently pressed to ensure water-tight attachment. The 8 GHz medical microwave (maximum wattage 20 W) was obtained from Emblation Microwave (Inglewood, Alloa, Scotland, UK).

### 4.2. Methods

#### 4.2.1. Preparation of Uric Acid Crystal Solution

Uric acid solutions were prepared at room temperature and at concentrations of 0.1 mg/mL and 0.2 mg/mL, similar to that found in humans with gout. Uric acid solutions of 20 mL were freshly prepared by dissolving 2 mg and 4 mg of powdered uric acid in 20 mL of deionized water before each experiment.

#### 4.2.2. Crystallization of Medium and Large l-Alanine Crystals

Large l-alanine crystals were crystallized at room temperature and the medium l-alanine crystals were crystallized using microwave heating [22]. In this regard, l-alanine solutions were prepared by dissolving 0.3 g of powdered l-alanine into 1.25 mL of distilled water in a 20 mL glass vial and stirred with a magnetic stir bar and heated to 60 °C. A 20 µL portion of the l-alanine solution was placed in the wells of the iCrystal plates. For medium sized l-alanine crystals, the solutions were heated at microwave power level 1 (900 W, conventional microwave oven) until all the liquid had been evaporated. For the large l-alanine crystals, the solutions in the iCrystal plates were left to crystallize at room temperature overnight.

#### 4.2.3. Decrystallization of the Small Uric Acid Crystals on the iCrystal Plates

**Figure 10:**
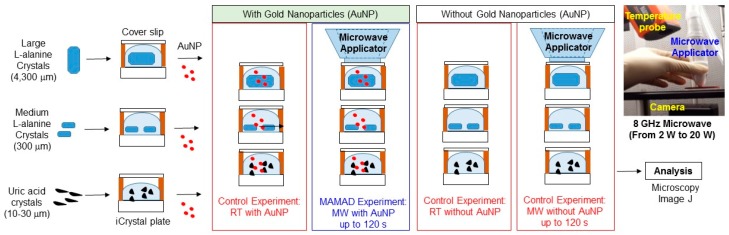
Schematic depiction of experimental procedures used for the decrystallization of uric acid and l-alanine crystals of various sizes using the MAMAD technique and control experiments.

Decrystallization of uric acid and l-alanine crystals were conducted under various conditions as shown in Figure 10. The smaller adhesive silicon isolator of diameter 3.5 mm was carefully attached to the PMMA platform and gently pressed to ensure secure attachment. A 5 µL portion of the uric acid solution was added to a well followed by 5 µL of the 20 nm gold nanoparticles solution (Au NP), and then the selected well was covered by a glass coverslip as also shown in Figure 10. The contents of the well (uric acid solution and Au NP) were exposed to various microwave wattages (2 W, 10 W, and 20 W) using an applicator tipped medical microwave at 10 s intervals for 60 s. The crystals in the wells were observed under an optical microscope with the glass coverslip removed (note: the optical microscope was stationed in close proximity to the microwave and so the delay time between microwave heating and capturing of the images took approximately 30 s). In the control experiments for uric acid, 5 µL of the uric acid solution and 5 µL of deionized water were added to a well and exposed to microwave wattages (2 W, 10 W, and 20 W) using applicator tipped medical microwave at 10-s intervals for 60 s. An additional control experiment was conducted in which 5 µL of the uric acid solution and 5 µL deionized water was added to a silicon well and left at room temperature. Optical images were taken every 10 s for 120 s. Each experimental and control experiments were conducted three times and the average data as well as data for selected individual trials are reported.

#### 4.2.4. Decrystallization of Medium and Large l-Alanine Crystals on the iCrystal Plates

Medium and large l-alanine crystals were selected based on the area of the well the crystal occupied: medium l-alanine crystals (~300 μm in length, 1–3 crystals) occupied less than half of the surface area of the well while the large l-alanine crystals (~4300 μm in length) occupied most of the surface area of the well. Large l-alanine crystals occupied one crystal per well due to the comparable size of the well and l-alanine crystals. In addition, 10 µL of gold nanoparticle solution was added to each well and then covered with a glass coverslip used to encase the well (the coverslip was removed to capture the optical images). As shown in Figure 10, the wells exposed to microwaves were exposed with the use of an applicator tipped medical microwave and observed under the optical microscope at 10 s increments for 120 s (there is an approximate 30 s delay between image captures). Microwave power used was: 2 W, 10 W, and 20 W. Each experiment was repeated at least three times. In the control experiments, 10 µL of deionized water were added to each well occupied by the l-alanine crystals. The crystals were then exposed to microwave power levels (2 W, 10 W, and 20 W) for 120 s. Optical images were taken every 10 s for 120 s. An additional control experiment was conducted whereby 10 µL deionized water was added to the l-alanine crystals in the well and left at room temperature. Optical images were taken every 10 s for 120 s. The control experiments were repeated at least three times and average data reported.

#### 4.2.5. X-ray Diffraction and ImageJ Analysis of Crystals

Powder XRD data was collected with Rigaku MiniFlex (The Woodlands, TX, USA). The total surface area and crystal count for each optical image of uric acid crystals were quantified and measured with the use of the ImageJ software (a free image processing and analysis software in Java). The crystal length and total surface area for all optical images of both medium and large l-alanine crystals were calculated using the ImageJ software. The area was obtained by closely outlining the selected crystals for each interval. An infrared thermometer was used to measure the temperature of the top layer of the solution that was exposed directly to the microwave heating. Each temperature measurement was completed within 3 s after the microwave heating stopped.

## 5. Conclusions

In this study, the MAMAD technique was demonstrated to be an effective method to significantly reduce the size and number of uric acid and l-alanine crystals. Our observations showed that microwave heating at 10 W in the presence of 20 nm gold nanoparticles were the most efficient experimental conditions for the decrystallization of uric acid and l-alanine crystals. Although small uric acid crystals and medium l-alanine crystals were decrystallized efficiently with the use of gold nanoparticles, decrystallization of large l-alanine crystals were found to be less efficient with the use of 20 nm gold nanoparticles, which can be attributed to the relative size of the large l-alanine crystals. The results in this study show that the MAMAD technique has the potential to be employed as a therapeutic aid for the decrystallization of crystals related to crystal deposition diseases.

## Supplementary Materials

Supplementary materials can be accessed at: http://www.mdpi.com/1420-3049/21/10/1388/s1.
