# Supplementary material for: Decrystallization of Crystals Using Gold “Nano-Bullets” and the Metal-Assisted and Microwave-Accelerated Decrystallization Technique
Source: Molecules. 2016 Oct 18;21(10):1388. doi: 10.3390/molecules21101388 (PMC5409504; doi:10.3390/molecules21101388)
Supplement: Supplementary file 1 [file molecules-21-01388-s001.pdf]

Supplementary Materials: Decrystallization of Crystals using Gold "Nano-bullets" and the Metal-Assisted and Microwave-Accelerated Decrystallization Technique

Nishone Thompson, Zainab Boone-Kukoyi, Raquel Shortt, Carisse Lansiquot, Bridgit Kioko, Enock Bonyi, Salih Toker, Birol Ozturk and Kadir Aslan

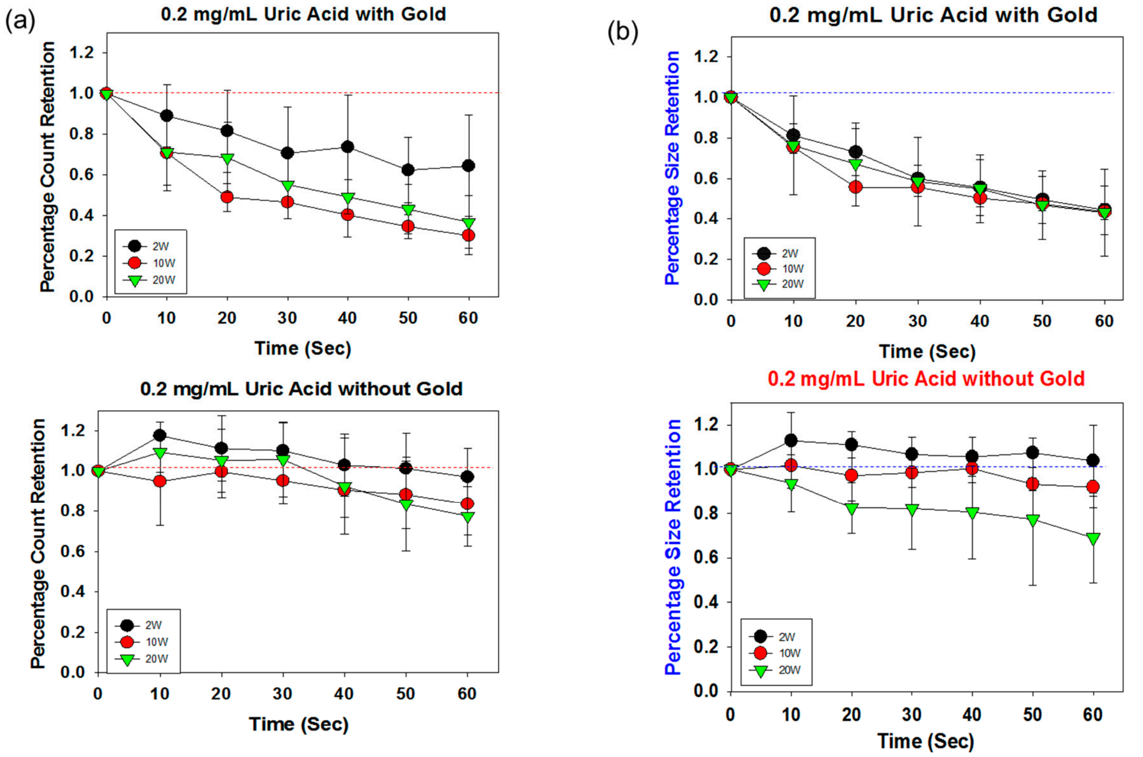

Figure S1. Normalized crystal (**a**) count and (**b**) size retention rate of small uric acid crystals in the presence of gold nanoparticles during 60 s of microwave heating. The initial concentration of uric crystals was set to 0.2 mg/mL.

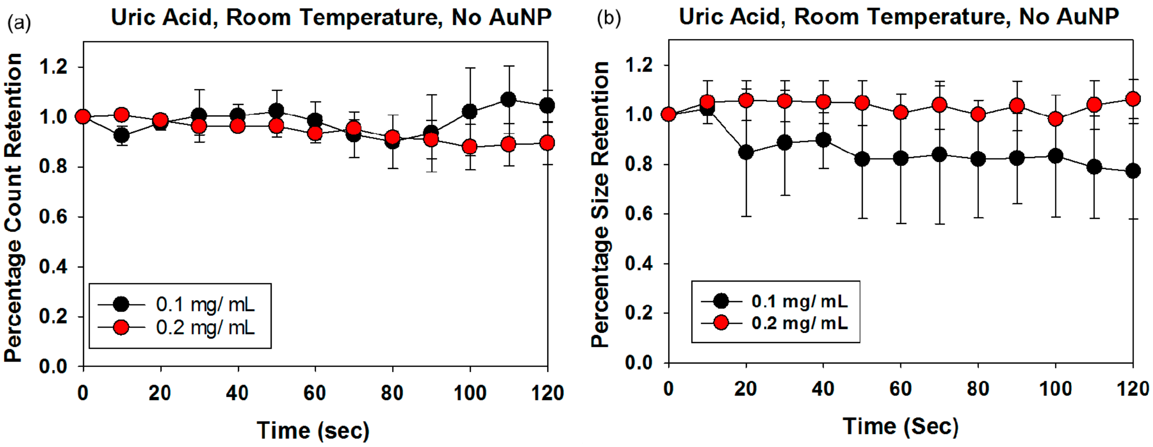

Figure S2. Normalized crystal (**a**) count and (**b**) size retention rate of uric acid crystals after 120 s of microwave heating in the presence of gold nanoparticles and in the absence of gold nanoparticles (control).

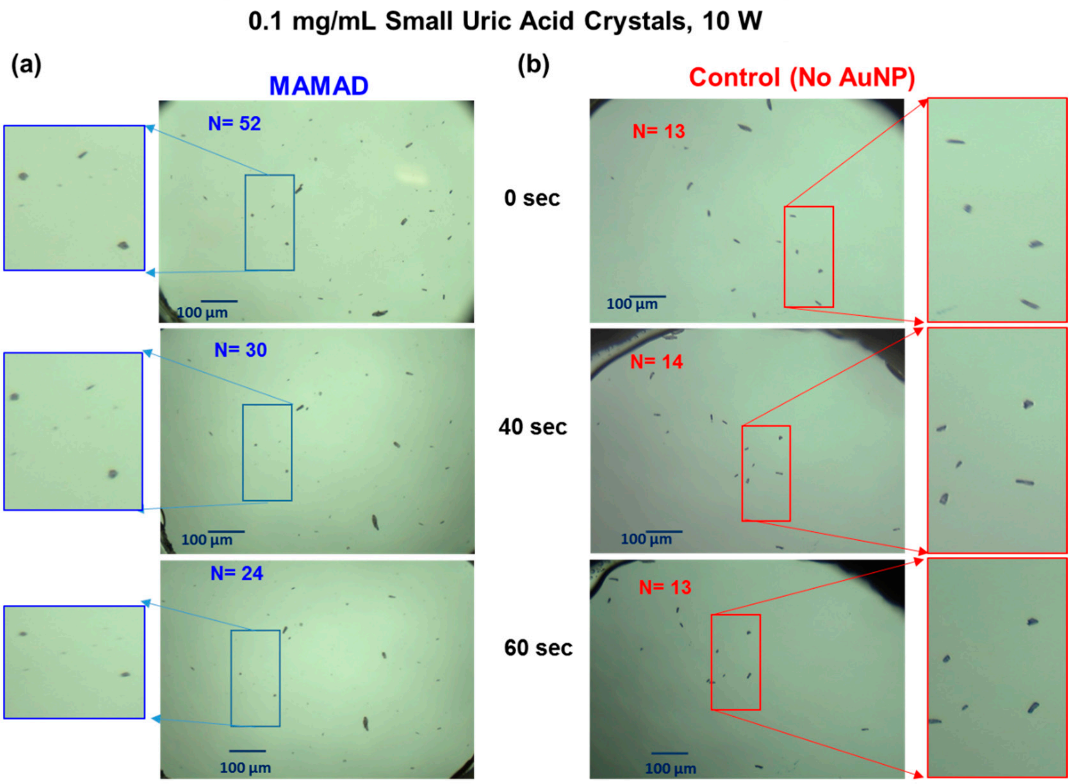

Figure S3. Optical images of small uric acid crystals (0.1 mg/mL) during 10 W microwave heating for 60 s (**a**) in the presence of gold nanoparticles (i.e., the MAMAD technique); (**b**) in the absence of gold nanoparticles.

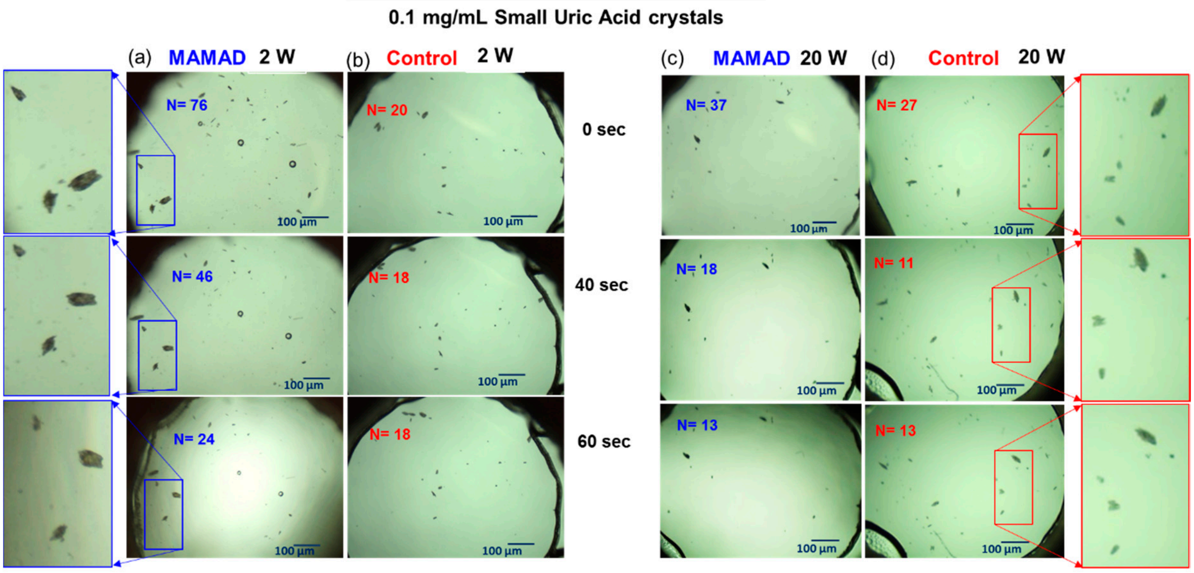

Figure S4. Optical images of small uric acid crystals (0.1 mg/mL) during microwave heating for 60 s (**a**) in the presence of gold nanoparticles at 2 W (i.e., the MAMAD technique); (**b**) in the absence of gold nanoparticles at 2 W (control); (**c**) in the presence of gold nanoparticles at 20 W (i.e., the MAMAD technique); (**d**) in the absence of gold nanoparticles at 20 W (control).

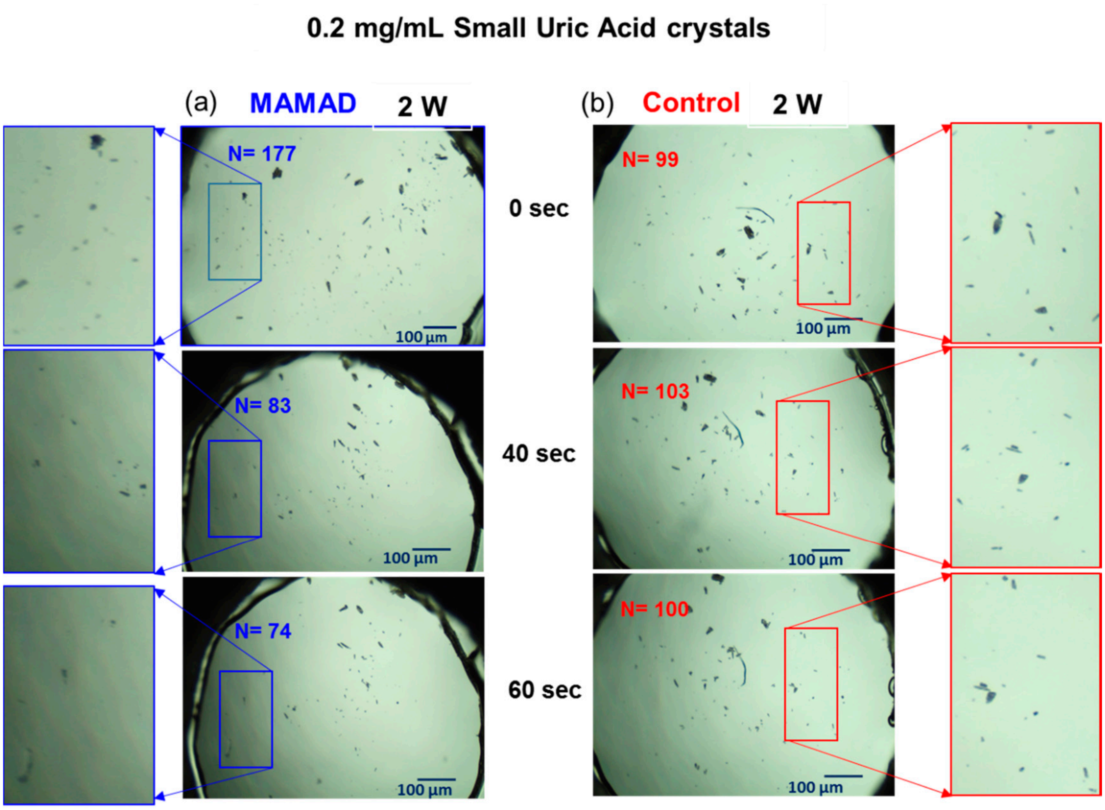

Figure S5. Optical images of small uric acid crystals (0.2 mg/mL) during 2 W microwave heating for 60 s (**a**); in the presence of gold nanoparticles (i.e., the MAMAD technique); (**b**) in the absence of gold nanoparticles.

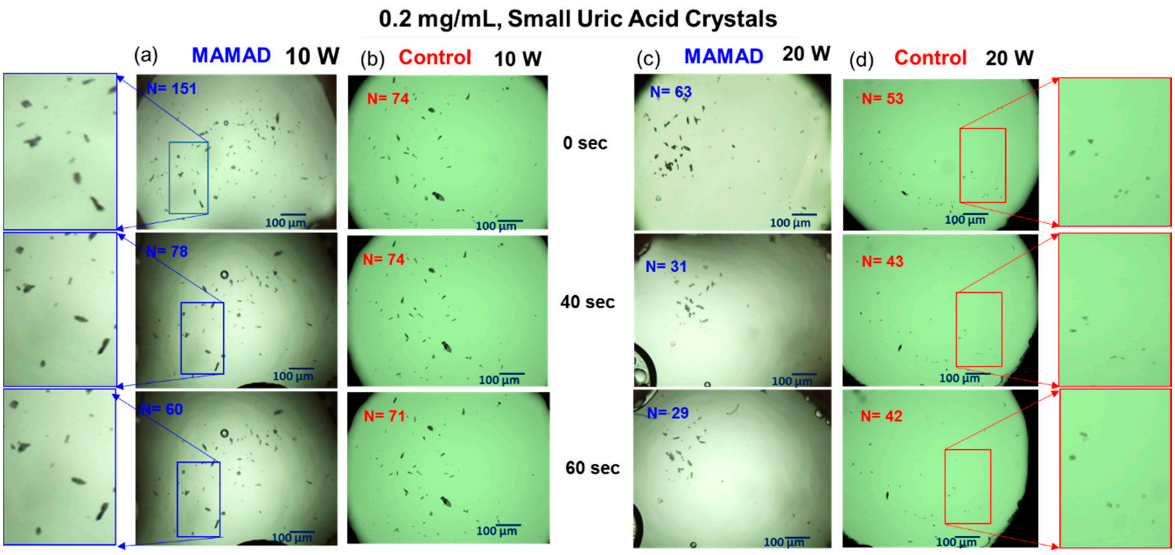

Figure S6. Optical images of small uric acid crystals (0.2 mg/mL) during microwave heating for 60 s (**a**) in the presence of gold nanoparticles at 2 W (i.e., the MAMAD technique); (**b**) in the absence of gold nanoparticles at 2 W (control); (**c**) in the presence of gold nanoparticles at 20 W (i.e., the MAMAD technique); (**d**) in the absence of gold nanoparticles at 20 W (control).

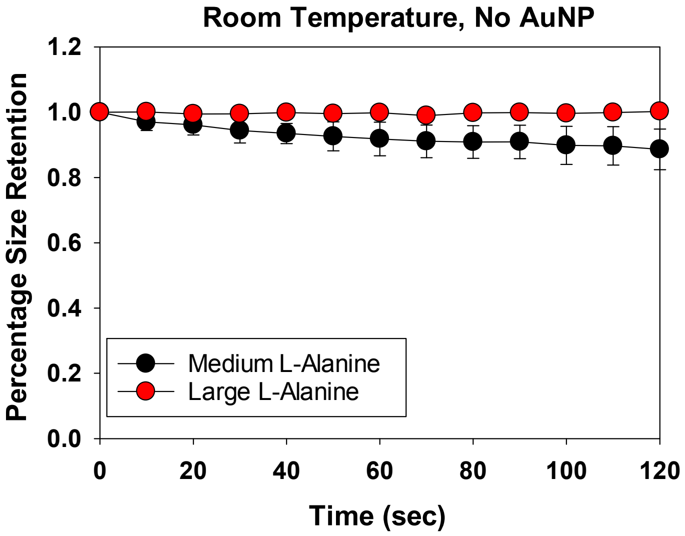

Figure S7. Reduction of crystal size at room temperature and without gold nanoparticles for medium and large L-Alanine crystals in deionized water.

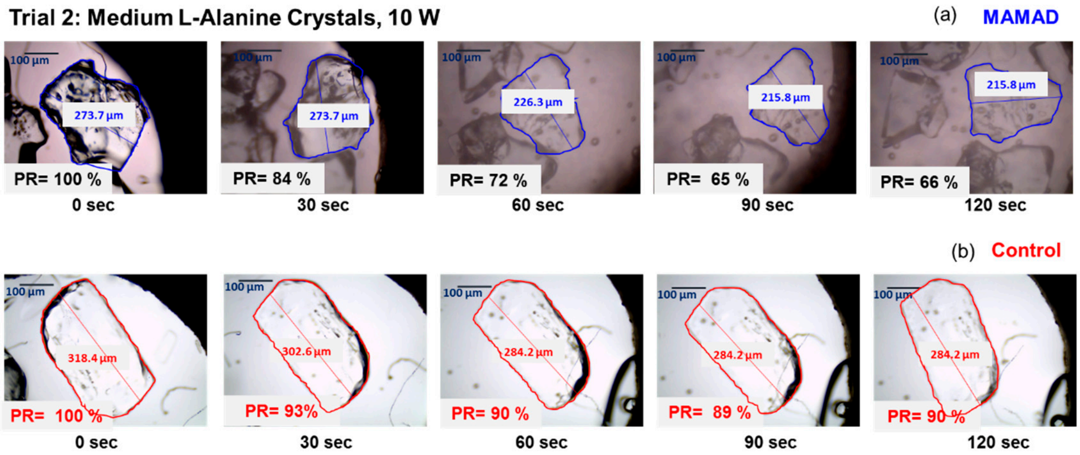

Figure S8. Trial 2**:** Optical images of medium-sized L-alanine crystals (initial size = 274 mm) during 10 W microwave heating for 120 s (**a**) in the presence of gold nanoparticles (i.e., the MAMAD technique); (**b**) in the absence of gold nanoparticles (control). PR = Percent Retention.

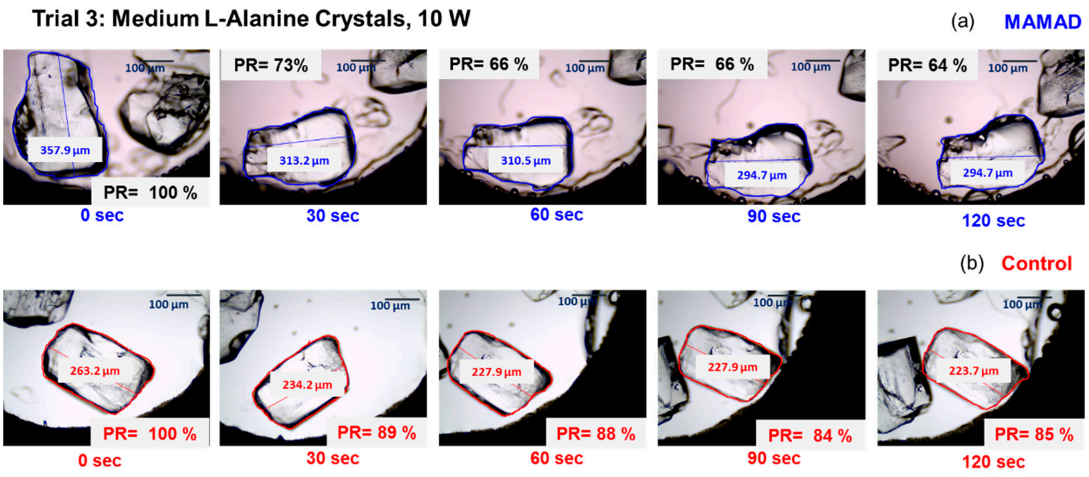

Figure S9. Trial 3**:** Optical images of medium-sized L-alanine crystals (initial size = 274 mm) during 10 W microwave heating for 120 s (**a**) in the presence of gold nanoparticles (i.e., the MAMAD technique); (**b**) in the absence of gold nanoparticles (control). PR = Percent Retention.

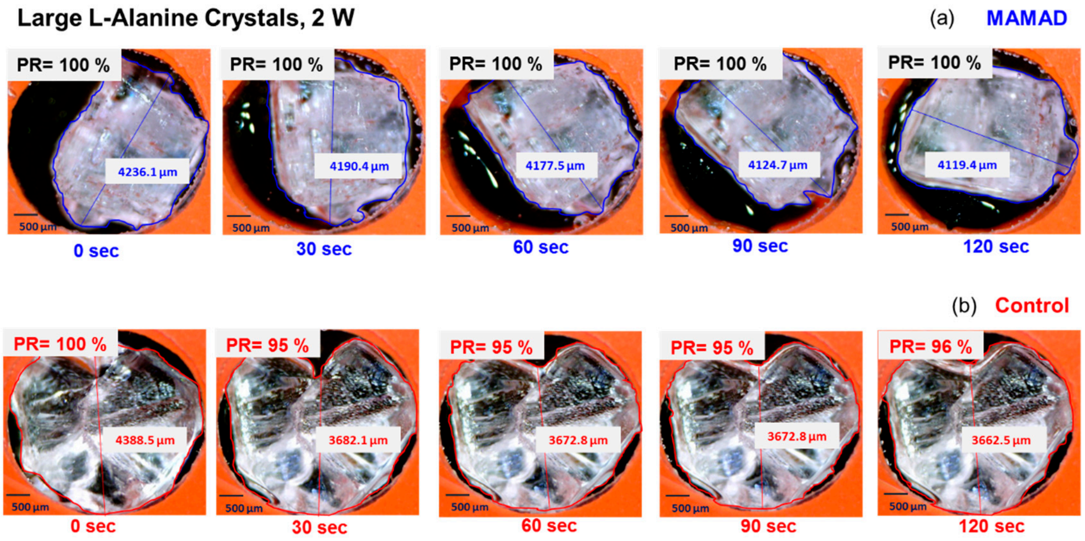

Figure S10. Optical images of medium-sized L-alanine crystals (initial size = 4236 mm) during 2 W microwave heating for 120 s (**a**) in the presence of gold nanoparticles (i.e., the MAMAD technique); (**b**) in the absence of gold nanoparticles (control). PR = Percent Retention.

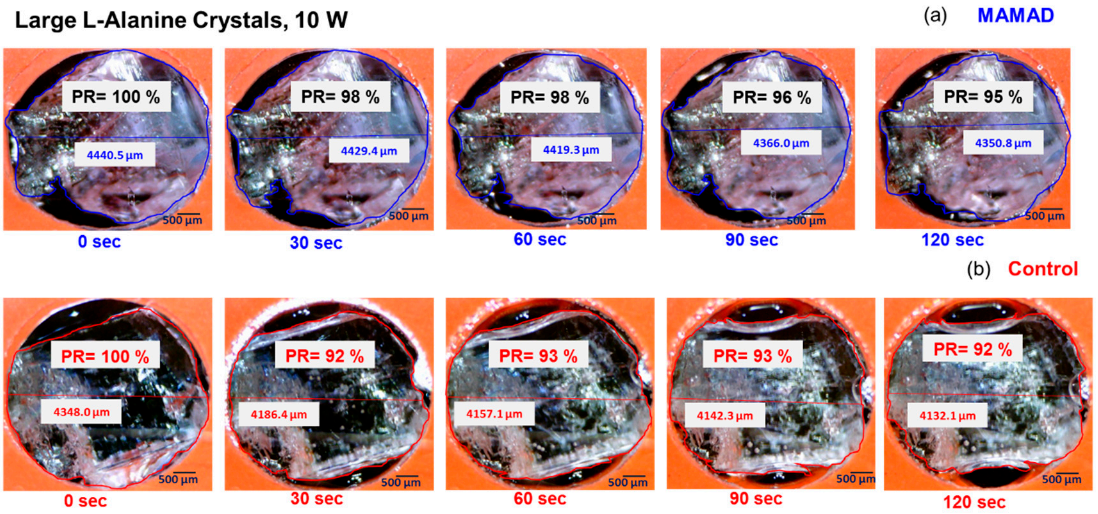

Figure S11. Optical images of medium-sized L-alanine crystals (initial size = 4441 mm) during 10 W microwave heating for 120 s (**a**) in the presence of gold nanoparticles (i.e., the MAMAD technique); (**b**) in the absence of gold nanoparticles (control). PR = Percent Retention.

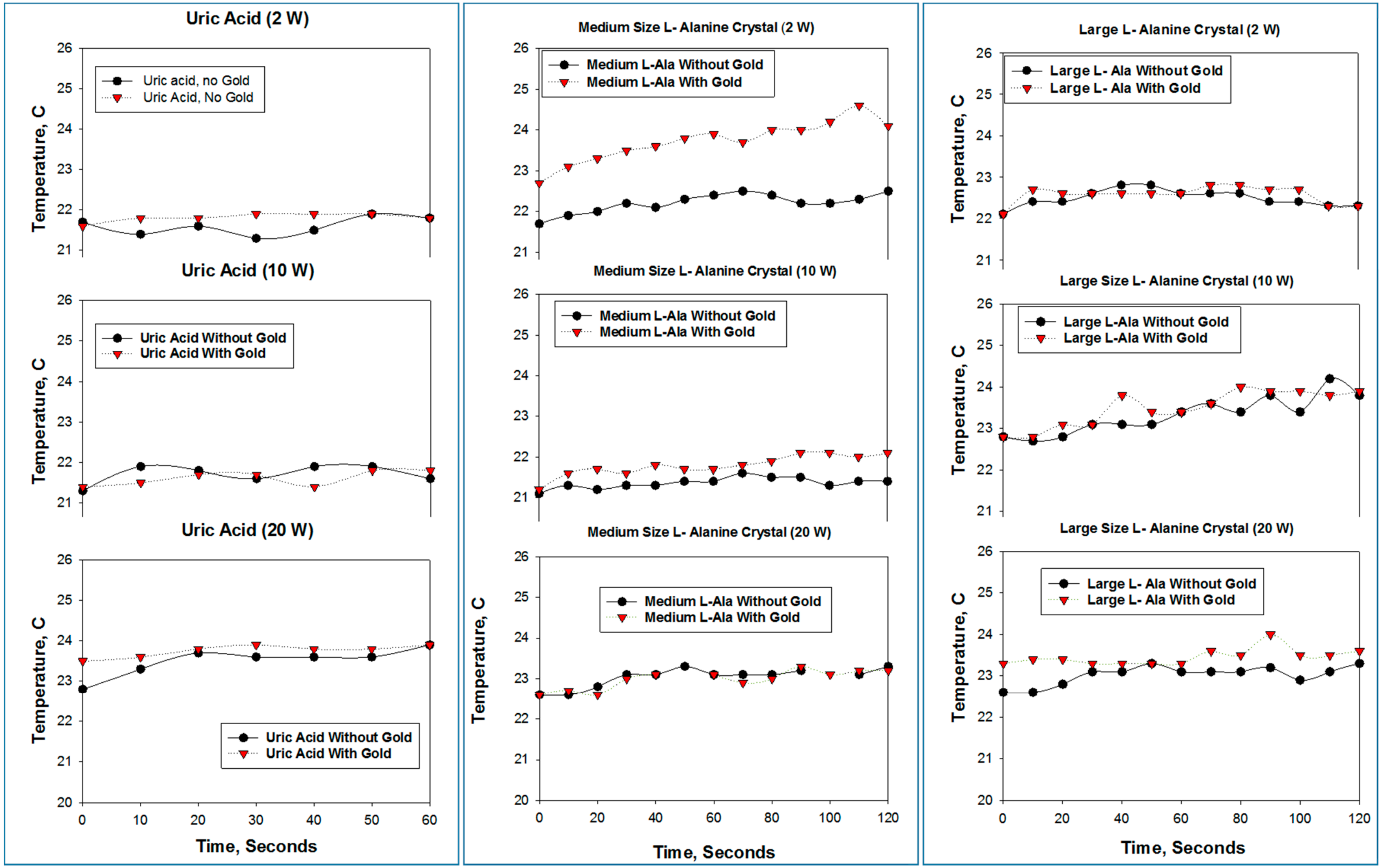

Figure S12. Real-time temperature measurements for uric acid crystals, medium and large L-alanine crystals during 2 W, 10 W and 20 W microwave heating for 60 s and 120 s, respectively in the presence of gold nanoparticles (i.e., the MAMAD technique) and in the absence of gold nanoparticles (control).
